# Performance Variability During Motor Learning of a New Balance Task in a Non-immersive Virtual Environment in Children With Hemiplegic Cerebral Palsy and Typically Developing Peers

**DOI:** 10.3389/fneur.2021.623200

**Published:** 2021-03-15

**Authors:** Minxin Cheng, Michael Anderson, Danielle E. Levac

**Affiliations:** ^1^Rehabilitation Games and Virtual Reality Laboratory, Department of Physical Therapy, Movement and Rehabilitation Sciences, Northeastern University, Boston, MA, United States; ^2^Department of Biology, Macalester College, St. Paul, MN, United States

**Keywords:** cerebral palsy, virtual reality, variability, motor learning, virtual environment, children

## Abstract

**Background:** Motor impairments contribute to performance variability in children with cerebral palsy (CP) during motor skill learning. Non-immersive virtual environments (VEs) are popular interventions to promote motor learning in children with hemiplegic CP. Greater understanding of performance variability as compared to typically developing (TD) peers during motor learning in VEs may inform clinical decisions about practice dose and challenge progression.

**Purpose:** (1) To quantify within-child (i.e., across different timepoints) and between-child (i.e., between children at the same timepoint) variability in motor skill acquisition, retention and transfer in a non-immersive VE in children with CP as compared to TD children; and (2) To explore the relationship between the amount of within-child variability during skill acquisition and learning outcomes.

**Methods:** Secondary data analysis of 2 studies in which 13 children with hemiplegic CP and 67 TD children aged 7–14 years undertook repeated trials of a novel standing postural control task in acquisition, retention and transfer sessions. Changes in performance across trials and sessions in children with CP as compared to TD children and between younger (7–10 years) and older (11–14 years) children were assessed using mixed effects models. Raw scores were converted to z-scores to meet model distributional assumptions. Performance variability was quantified as the standard deviation of z-scores.

**Results:** TD children outperformed children with CP and older children outperformed younger children at each session. Older children with CP had the least between-child variability in acquisition and the most in retention, while older TD children demonstrated the opposite pattern. Younger children with CP had consistently high between-child variability, with no difference between sessions. Within-child variability was highest in younger children, regardless of group. Within-child variability was more pronounced in TD children as compared to children with CP. The relationship between the amount of within-child variability in performance and performance outcome at acquisition, retention and transfer sessions was task-specific, with a positive correlation for 1 study and a negative correlation in the other.

**Conclusions:** Findings, though preliminary and limited by small sample size, can inform subsequent research to explore VE-specific causes of performance variability, including differing movement execution requirements and individual characteristics such as motivation, attention and visuospatial abilities.

## Introduction

Cerebral palsy (CP) is the leading cause of physical disability in childhood (1–4). Unilateral spastic CP, or hemiplegia, is the most common subtype, representing 35–40% of new diagnoses (4, 5). Children with hemiplegia have motor, cognitive, sensory and perceptual challenges that limit postural control and activities of daily living, reducing functional independence (6–10). Assisting children to learn new motor skills, improve existing skills, and transfer skills to enhanced function in the real world is a primary goal of rehabilitation (11–13). However, much remains to be understood about motor learning impairments in children with CP (11–13).

Information-processing, attention, motor planning, and motor execution impairments can differ in children with CP as compared to typically developing (TD) peers, influencing the rate and extent of motor learning (12, 13). While children with CP improve in new motor task learning with practice (14–16), they may require greater duration of practice to achieve competency, while demonstrating lower accuracy and greater variability in task performance outcomes over repeated trials as compared to TD peers (14, 16–25). The heterogeneous nature of motor and cognitive impairments in CP allows for significant variability in performance outcomes in children of the same age and Gross Motor Function Classification System Level (24, 26–30).

Variability, traditionally conceptualized as the opposite of stability, is a fundamental characteristic of human performance (31, 32). Sternad defines variability as an umbrella term for “all sets or series of observations that are non-constant” (32). Variability is usually reduced with practice of a new motor skill. A prevalent view is that variability impedes the accuracy and precision required for skill attainment (31, 32). In contrast, some amount of variability may be beneficial to support the search for optimal solutions in differing task conditions (33–35). Indeed, Hadders-Algra defines variability as “the capacity to select from the repertoire the motor strategy that fits the situation best” (36). Ranganathan et al. (31) relate this view of variability to behavioral flexibility, which they define as “the ability to achieve the same task outcome using different movement solutions.” Whether adaptive or detrimental, variability can occur at the level of task performance (in performance outcomes) and at the level of movement execution (in kinematic strategies used to achieve the outcome). Exploring both within-child (e.g., variability across different timepoints) and between-child variability (e.g., variability between children at the same timepoint) at both task and movement levels is important to understand differences in children's responses to interventions (37).

Movement execution variability in children with CP is highly correlated with severity of motor impairment (38, 39). Children with CP may demonstrate more movement execution variability because of challenges suppressing normal intrinsic motor system noise (40, 41). Their motor learning impairments may also affect the formation of internal models of movement and the interpretation of feedback mechanisms that could reduce variability (42). Movement execution variability has been investigated in gait (40, 43) and speech kinematics (44–46) in this population. For example, children with CP have a higher stride to stride variability, with more variation in muscle synergies during walking (39). In speech kinematics, Chen et al. (45) found longer coefficients of variation of utterance duration for short speech tasks in children with CP as compared to TD children, with greater variability as task complexity increased. However, movement execution variability in children with CP can decrease with training. For example, interventions in which children adapt to different gait speeds in each leg using a split-belt treadmill can decrease stride to stride variability in children with hemiplegic CP in ways that are significantly correlated with learning improvements (41).

Non-immersive virtual environments (VEs) in which children use body movements to interact with virtual objects displayed on a 2-dimensional (2D) flat-screen display are pediatric rehabilitation interventions that can support motor skill improvement and motor learning (47). There is strong evidence for the effectiveness of VE-based interventions to improve upper extremity functioning (48–51) and postural control (52–54) in children with hemiplegic CP. The unique practice conditions of non-immersive VEs may impact movement execution and performance variability. For example, interactions with virtual objects involve differing perceptual-motor affordances as compared to interaction with objects in the physical environment (55, 56). A lack of 3D depth cues in a non-immersive VE influences distance estimates of where objects are in space, which may increase uncertainty about movement accuracy. In addition, hand-held peripheral controllers that track movement (such as the one required by the Nintendo Wii or the HTC VIVE) may influence task interaction (56) as opposed to direct motion tracking.

Several studies have explored changes in movement execution variability in children with CP who learn a seated reaching task in a non-immersive VE as compared to in a physical environment. Children reduce their movement execution variability in repeated training in non-immersive VE, with some kinematic improvements transferring to improved performance of the same movement in the physical environment (57). Robert et al. (16) undertook reach-to-grasp training in both physical and 2D flat-screen VEs in children with CP, finding similar improvements in kinematic variables in both training groups. Robert and Levin (58) compared reaching movement kinematics in 2D virtual reality and physical environment in typically developing children and children with CP. Several kinematic variables differed between reaches in the VE and the physical environment for children with CP, with only small clinically insignificant differences between CP and TD children. Overall, children moved more slowly in the VE. When children with CP use a hand-held game controller to interact with a non-immersive VE, they demonstrate both within- and between-child variability in terms of upper-extremity movement patterns used to play a single 2D active video game (59, 60). Some of this variability may be explained by personal and predisposing factors, such as gender, experience with video game play, and upper extremity impairment level (52).

Less is known about task performance variability in non-immersive VEs. Exploring this issue is important to contribute to the ongoing discussion about variability as both an adaptive and detrimental characteristic of motor learning (31, 32). Greater knowledge about within- and between-child variability in performance can provide new information relevant to conclusions about intervention effectiveness and inform sample size considerations for clinical trials. With greater understanding of variability in new task learning in non-immersive VEs, we can better guide therapists who endeavor to adhere to motor learning principles underlying experience-dependent neuroplasticity in rehabilitative strategies (61). For example, decisions about practice dosage, amount of repetition, and timing of progression of difficulty and challenge levels are often made on the basis of consistent performance improvements (i.e., a lack of variability). Understanding children's variability in performance over time is important because non-immersive VEs are often used as home intervention programs (62) and are not directly supervised by therapists; therefore, they cannot observe children's performance to understand how they perform over repeated trials.

The purpose of this study is to (1) Quantify within- and between-child performance variability in motor skill acquisition, retention and transfer in a non-immersive VE in children with hemiplegic CP and TD children; and (2) Explore the relationship between the amount of within-child variability during skill acquisition and retention performance. We hypothesize that children with CP will demonstrate greater between- and within-child variability than TD children, that younger children will demonstrate greater variability as compared to older children, and that variability will differ by learning stage and demonstrate a relationship with learning outcomes.

## Study Design and Methods

**Figure 1:**
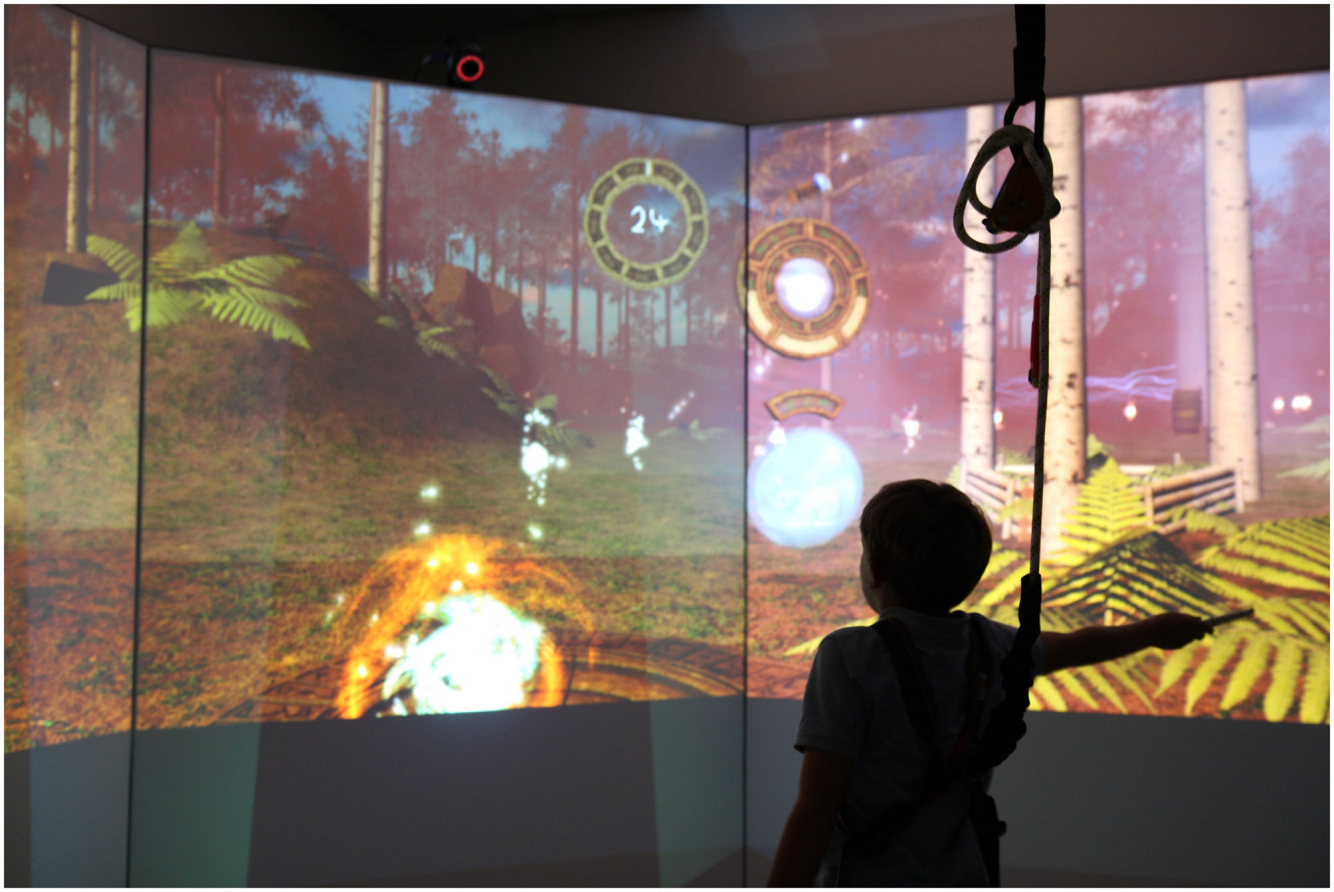
Displays the Study 1 virtual environment, showing the path emerging in front of the participant (the white dots).

**Figure 2:**
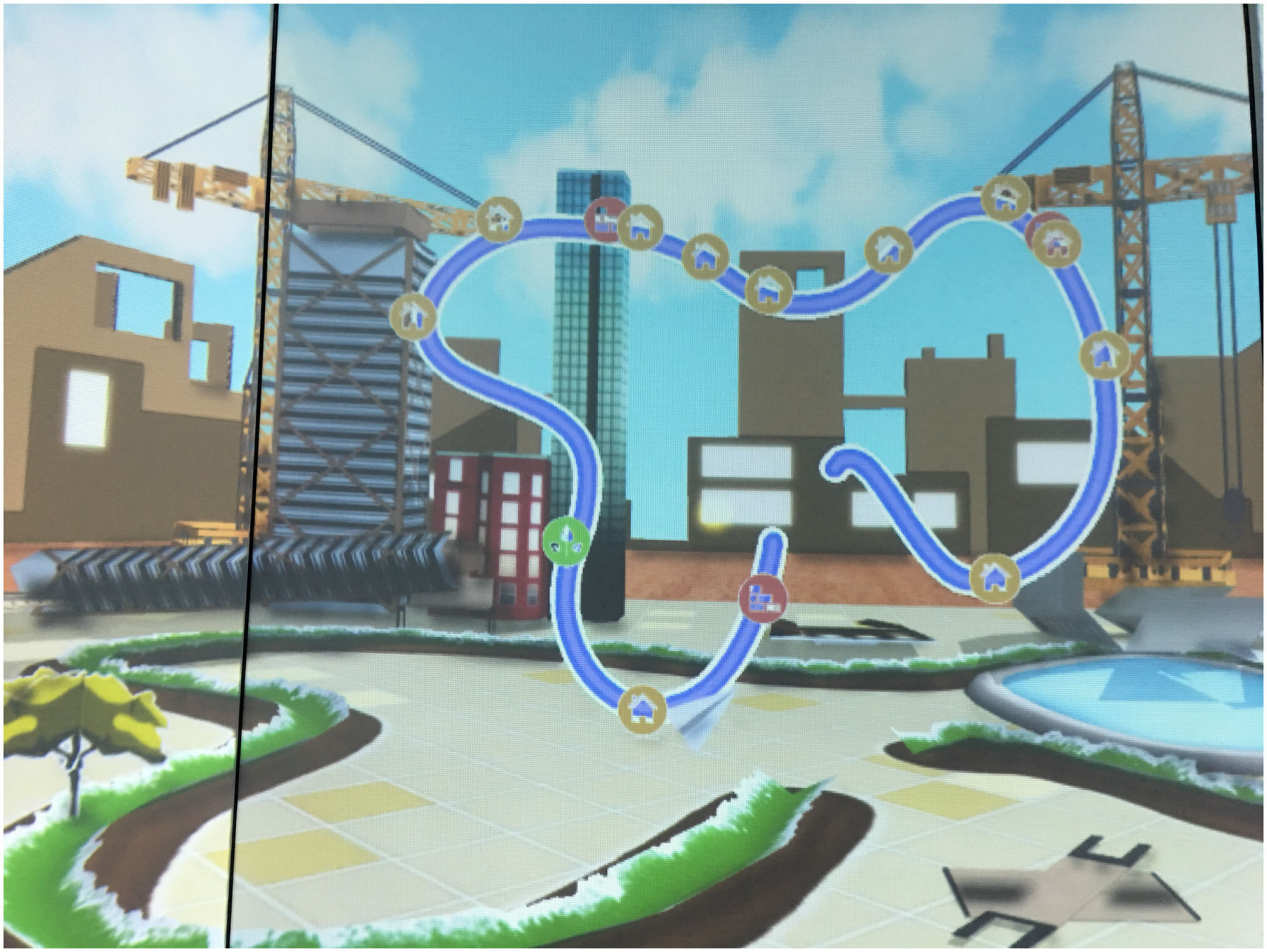
Displays the Study 2 virtual environment, showing the entire path visible to the participant (the blue line).

We undertook a secondary data analysis of 2 studies undertaken in our lab in which children with hemiplegic CP at Gross Motor Function Classification System (GMFCS) Levels I and II and typically developing children acquired one of 2 new balance skills in a non-immersive VE [the Stability and Balance Learning Environment (STABLE; Motekforce Link, The Netherlands), a 130 degree projection flat-screen VE in which interaction is via a force plate and motion capture cameras]. Forty-seven children participated in Study 1 and 33 children participated in Study 2. All children undertook baseline postural control tests (eyes closed stance, single leg stance, tandem stance, and mediolateral and anteroposterior limits of stability) on the STABLE prior to beginning the task. In both Study 1 and Study 2, children practiced a task requiring them to move their center of pressure (CoP) within a static base of support to control a virtual avatar (Acquisition). Children used CoP movements to control the avatar to follow a predetermined path displayed in the non-immersive VE as closely as possible. In Study 1 (Figure 1), the avatar moved along a path in a first-person perspective such that view of the path in the VE emerged according to the children's movements. In contrast, in Study 2 (Figure 2); the full path was always visible to the child in a 3rd person perspective. In both studies, VE visual and auditory feedback changed according to children's movements. The tasks are described in more detail in (63, 64). Acquisition involved 20 trials of practice; children returned 2–7 days after acquisition for a retention and transfer session, in which they performed the task in the same condition as acquisition (Retention; 10 trials) and in a more motorically-challenging condition (Transfer; 10 trials).

## Analyses

Changes in performance score across trials and sessions, as well as differences in performance between children with CP and TD children, and children differing in age (7–10 vs. 11–14 years), were assessed using mixed effects models via the lme4 package in R version 3.6.0. Raw performance scores were converted to z-scores to more closely meet distributional assumptions of the models. A z-score of 0 represents mean performance. Model selection was conducted using an information-theoretic approach based on Akaike's Information Criterion (AIC) (65) [as described in (66)]. Briefly, a set of models is generated based on explanatory variables and higher-order effects (e.g., interactions and/or polynomial terms) of interest. A reduced set of explanatory variables is selected, to prevent overfitting and determine the most important effects in the model. The reduced model is selected using AIC, in which models are ranked in increasing order by AIC values, which are then used to calculate “Akaike weights” for each model. These are commonly interpreted as the probability that the given model is the “best” in the set in terms of minimizing loss of Kullback-Leibler (66) information, providing a straightforward means of comparing relative model fits.

Models in our initial comparison set included both least-square and mixed effects implementations. The full set of explanatory variables and higher-order effects tested included trial number (linear only vs. second-order polynomial), group (TD vs. CP), age group (7–10 vs. 11–14 years), and interactions between group and both age group and the polynomial term for trial. For the top selected models, study was also added as an explanatory variable, as both a main effect and interaction with group and age group, in order to quantify the size of the difference between studies compared with other sources of variation.

Mixed models initially included random parameters for both slope and intercept across trials for each subject. However, for models that included random slope parameters, the numerical search method failed to converge on a maximum-likelihood solution, likely as a result of our relatively small sample size, particularly for the CP group. Mixed-effects implementations of our comparison models therefore only included a random intercept parameter. Differences in mean z-scores among sessions were examined separately using a mixed-effects model that included group, age group, and session as fixed effects, and a random intercept term for subject. Pair-wise differences between groups were examined using Tukey-adjusted *post-hoc* comparisons of estimated marginal means in the “emmeans” package in R.

Because AIC and statistics derived from it only assess relative fit among different models, absolute model fit was also assessed using *R*^2^ values calculated from model deviances using the r.squaredGLMM command from the MuMIn package in R. This generates a “marginal” *R*^2^ which expresses the variance explained by the fixed effects only, as well as a “conditional” *R*^2^ that reflects the variance explained by the whole model (fixed + random factors).

Between-child variability was quantified as the standard deviation (SD = square root of the variance) for each of the 4 groups. We quantified within-child variability as the SD of the trial-to-trial difference in individual z-scores (sdDiff). We tested equality of variance using Levene's test between group and age group among sessions and at each session. Mean sdDiff was compared among sessions, and between groups and age groups, using ANOVA followed by Tukey-adjusted *post-hoc* tests. To examine the relationship between within-child variability (sdDiff) in a session and 2 performance outcomes of that session (maximum z-score and the mean z-score) we ran a multiple regression model for each correlation per study, with group as an explanatory variable, both as a main effect and as an interaction with the performance measure.

## Results

### Performance Differences Between Children With CP and TD Children and Between Older and Younger Children at Each Session

Table 1 provides the mean and SD of z-scores for each age group across sessions and at each session. Table 2 presents the mean and SD of baseline postural control tests for each age group. Table 3 presents the effect sizes [Cohen's d (67) and Hedges g (68)] for each age group at each session.

**Table 1:** Mean and SD of Z-scores for each age group across sessions and within each session.

**Group**	**Age group**	**N**	**Mean (SD) Z score**	**Session**	**Mean (SD) Z score**
TD	7–10 yr	41	−0.090 (SD 0.908)	Acquisition	−0.307 (SD 0.914)
				Retention	0.249 (SD 0.891)
				Transfer	0.056 (SD 0.773)
	11–14 yr	26	0.427 (SD 0.900)	Acquisition	0.141 (SD 0.917)
				Retention	0.909 (SD 0.644)
				Transfer	0.529 (SD 0.856)
CP	7–10 yr	8	−1.308 (SD 0.970)	Acquisition	−1.36 (SD 1.059)
				Retention	−1.084 (SD 0.666)
				Transfer	−1.404 (SD 0.985)
	11–14 yr	8	−0.602 (SD 0.705)	Acquisition	−0.718 (SD 0.589)
				Retention	−0.287 (SD 0.895)
				Transfer	−0.529 (SD 0.759)

**Table 2:** Mean and SD of baseline postural control tests for each age group.

**Group**	**Age group**	Mean (SD) ML^*^ left	Mean (SD) ML^*^ right	Mean (SD) AP^*^ anterior	Mean (SD) AP^*^ posterior	Mean (SD) LOS^*^
TD	7–10 yr	10.9543 (SD 3.9969)	11.3786 (SD 3.0423)	6.6171 (SD 3.9413)	6.9171 (SD 1.6482)	13.1769 (SD 1.4142)
	11–14 yr	11.5133 (SD 3.1909)	12.5633 (SD 3.5228)	8.9383 (SD 2.6421)	4.5200 (SD 1.4119)	12.1173 (SD 2.3520)
CP	7–10 yr	13.9318 (SD 2.4724)	13.4954 (SD 2.8786)	8.4153 (SD 2.7611)	7.2518 (SD 2.3917)	15.4966 (SD 5.9644)
	11–14 yr	13.2888 (SD 2.2055)	13.8192 (SD 2.1315)	8.9438 (SD 3.0108)	8.0188 (SD 1.8754)	13.9663 (SD 1.7177)

* *ML, Medio-lateral excursion; AP, Anterior-posterior excursion; LOS, Limits of stability*.

**Table 3:** Effect sizes for between-group differences at each session.

**Session**	**TD-CP**	**TD-CP**	**TD-CP**	**CP 7–10 yr - CP 11–14 yr**	**TD 7–10 yr – TD 11–14 yr**
	**All**	**7–10 yr**	**11–14 yr**		
Acquisition	Cohen's d = 0.947 Hedges g = 0.940	Cohen's d = 1.127 Hedges g = 1.109	Cohen's d = 1.003 Hedges g = 1.980	Cohen's d = 0.752 Hedges g = 0.711	Cohen's d = 0.490 Hedges g = 0.484
Retention	Cohen's d = 1.444 Hedges g = 1.431	Cohen's d = 1.547 Hedges g = 1.522	Cohen's d = 1.694 Hedges g = 1.654	Cohen's d = 1.010 Hedges g = 0.955	Cohen's d = 0.820 Hedges g = 0.811
Transfer	Cohen's d = 1.456 Hedges g = 1.442	Cohen's d = 1.806 Hedges g = 1.777	Cohen's d = 1.256 Hedges g = 1.226	Cohen's d = 0.996 Hedges g = 0.941	Cohen's d = 0.586 Hedges g = 0.579

**Figure 3:**
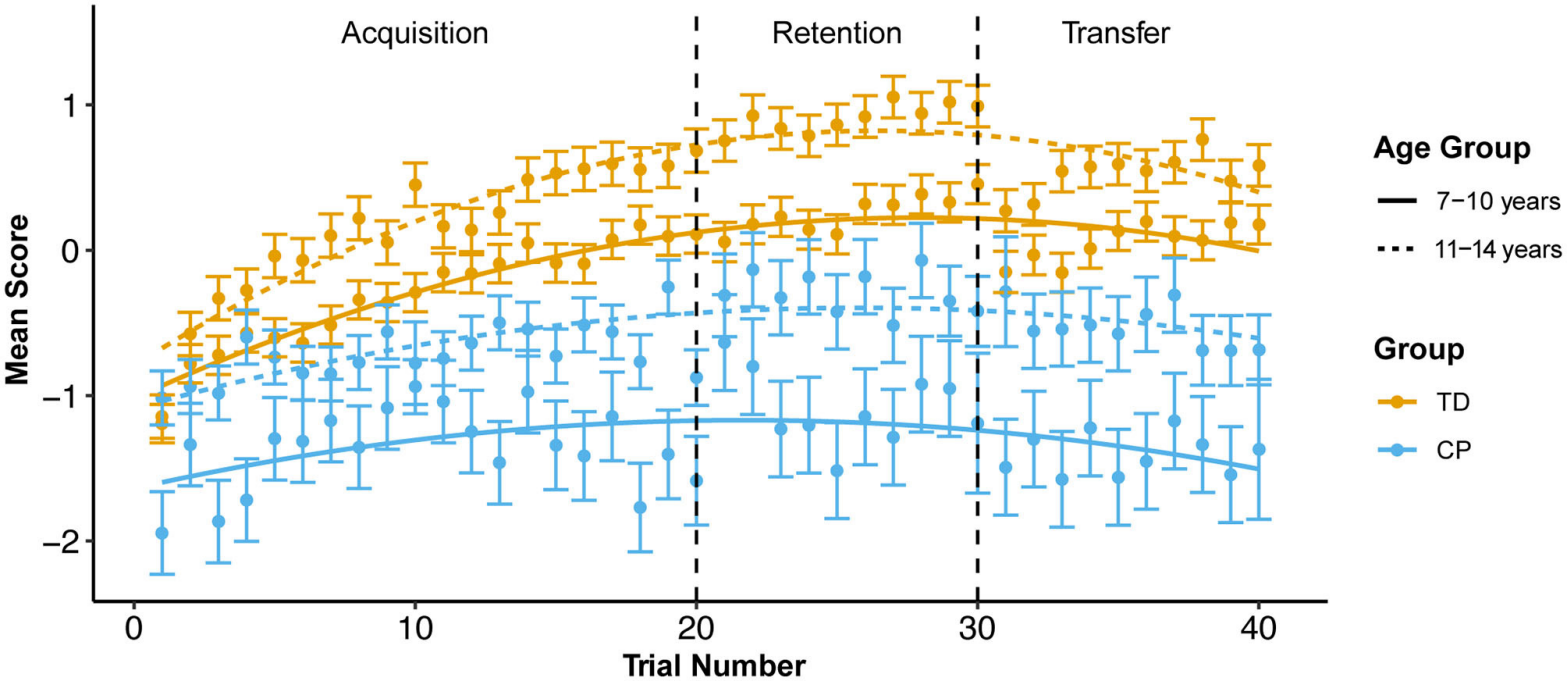
Mean z-score by group and age group across all trials at each session. Error bars use sdDiff (the standard deviation of the pair-wise trial-to-trial differences). SE is calculated by devising the SD by the square root of the number of subjects in each trial.

Figure 3 illustrates that children with CP have consistently lower scores as compared to TD children (*t* = −7.102, *p* < 0.001, estimate (CP) = −1.015, mean difference = 0.809), while the performance for younger participants is consistently below that for older participants (*t* = 4.604, *p* < 0.001, estimate (older) = 0.5206, mean difference = 0.780). Across sessions, mixed effects models show that the largest effects are associated with trial (*t* = 21.027, *p* < 0.001), with a positive linear effect indicating that most participants improve over time. However, the negative non-linear effect indicates this tendency toward improvement tends to diminish or even reverse as trial number increases within a session. For example, in the acquisition session, performance of the younger participants with CP drops off pronouncedly at the end of the session. Within age and group, the relationship with trial varies widely among participants, ranging from linear (both positive and negative slopes) to unimodal. The models show that the *R*^2^ almost doubles (*R*^2^ = 0.304 vs. *R*^2^ = 0.541) when accounting for these effects of random between-child variation.

### Between-Child Variability Across Sessions and Per Session: Group (TD vs. CP) and Age (Younger vs. Older) Differences

There is a significant difference in the amount of between-child variability between children with CP and TD children across all sessions [*F*_(1, 81)_ = 9.254, *p* < 0.001, mean difference = −1.071]. Younger children with CP demonstrate the most between-child variability, while older children with CP have the least [*F*_(3, 81)_ = 1.888, *p* < 0.001, mean difference = −0.706]. For TD children, there is no difference in the amount of between-child variability between younger and older children [*F*_(1, 65)_= 1.018, *p* = 0.758, mean difference = −0.517]. Older TD children and children with CP differ significantly in amount of between-child variability [*F*_(1, 47)_ = 1.627, *p* < 0.001, mean difference = 1.023]. There is no difference in between-child variability between the 3 sessions in younger children with CP [*F*_(2, 5)_ = 0.568, *p* = 0.568], while younger TD children demonstrated significant differences between sessions [*F*_(2, 23)_ = 5.375, *p* = 0.005].

Older TD children show significant differences [*F*_(2, 38)_ = 11.843, *p* < 0.001] between sessions, with the highest variability during acquisition, lowest during retention, and an increase again during transfer. Older children with CP also show significant differences [*F*_(2, 5)_ = 9.924, *p* < 0.001], but opposite to the pattern in the older TD children: lowest variation during acquisition, highest during retention, then a decrease during transfer. Younger TD children show significant differences [*F*_(2, 23)_ = 5.375, *p* = 0.005], with another distinct pattern: a peak in between-child variability during acquisition, followed by a steady decrease throughout retention and transfer. Younger children with CP do not show significant differences [*F*_(2, 5)_ = 0.568, *p* = 0.568] in variability between sessions. There are no significant differences in between-child variability between TD children and children with CP or between younger or older children in the transfer session.

### Within-Child Variability Across Sessions and Per Session: Group (TD vs. CP) and Age (Younger vs. Older) Differences

The strongest difference in within-child variability was associated with age, with younger children demonstrating greater within-child variability as compared to older children when pooled across sessions and group (*t* = 3.3, *p* = 0.001, mean difference = 0.157). Children with CP showed a non-significant trend toward lower within-child variability as compared to TD children (*t* = 1.9, *p* = 0.06, mean difference = −0.131). Within-child variability did not differ significantly among sessions (*p* > 0.1). When comparing across groups and age groups within each individual session, there were no significant differences, possibly due to high variability among individuals and low sample sizes. Older TD children displayed a trend toward higher within-child variability as compared to older children with CP in the acquisition session (*t* = 1.7, *p* = 0.10, mean difference = 0.237) and younger children with CP in the transfer session (*t* = 1.7, *p* = 0.09, mean difference = 0.216).

Table 4 provides between- and within-child variability for each age group overall and for each age group at each session.

**Table 4:** Within- and between-child variability for each age group and for each age group at each session.

**Group**	**Age group**	Mean (SD) between-child variability^*^	Mean (SD) within-child variability^**^	**Session**	Between-child variability^*^	Within-child variability^**^
TD	7–10 yr	−0.090 (SD 0.908)	0.840 (SD 0.344)	Acquisition	0.914	0.841
				Retention	0.891	0.835
				Transfer	0.773	0.843
	11–14 yr	0.427 (SD 0.900)	0.700 (SD 0.368)	Acquisition	0.917	0.797
				Retention	0.644	0.564
				Transfer	0.856	0.734
CP	7–10 yr	−1.308 (SD 0.970)	0.774 (SD 0.440)	Acquisition	0.106	0.867
				Retention	0.666	0.859
				Transfer	0.985	0.582
	11–14 yr	−0.602 (SD 0.705)	0.527 (SD 0.269)	Acquisition	0.589	0.560
				Retention	0.895	0.438
				Transfer	0.759	0.561

* *SD of z-score*.

** *sdDiff (SD of the pair-wise trial-to-trial differences per child)*.

**Figure 4:**
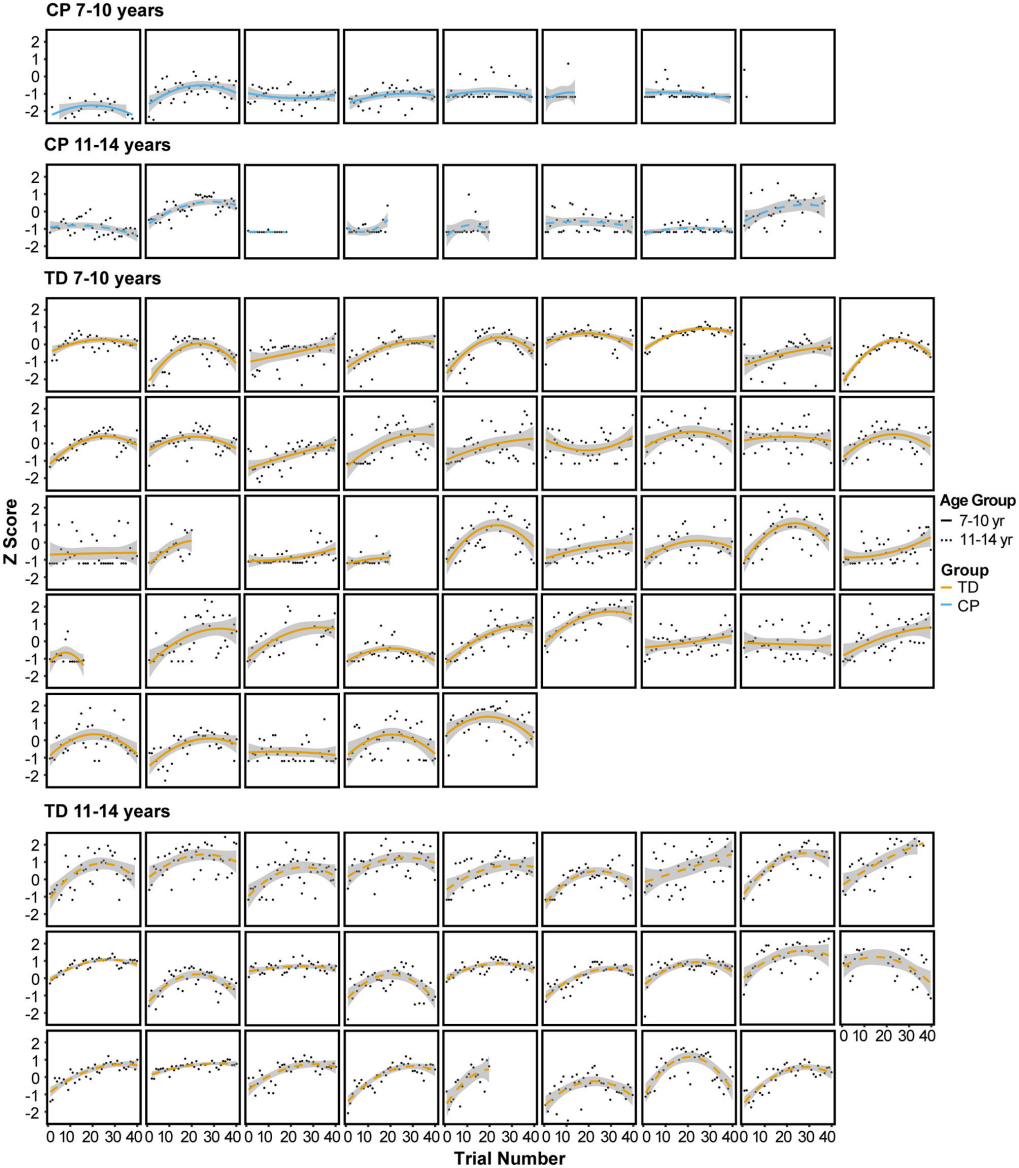
Performance score for each individual participant across all trials of the 3 sessions, fit with a quadratic curve.

Figure 4 illustrates the performance score for each individual participant across all trials of the 3 sessions, fit with a quadratic curve.

### Relationship Between the Amount of Within-Participant Variability in Acquisition and Performance Outcomes at Each Session

These analyses revealed a strong effect of study as an explanatory variable. In Study 1, there was a significant positive relationship of sdDiff during acquisition with MaxZ score at acquisition (*R*^2^ = 0.485, *p* < 0.001); this relationship did not differ between TD children and children with CP. There was no significant relationship with MeanZ. The amount of variability (sdDiff) in acquisition is not significantly correlated with MaxZ or MeanZ in retention or transfer sessions for Study 1.

For study 2, there was no significant negative relationship of amount of variability in acquisition with maxZ in acquistion (*R*^2^ = 0.237, *p* = 0.100). There was a significant negative relationship of amount of variability in acquisition with meanZ (*R*^2^ = 0.442, *p* < 0.001) in acquisition, with no difference between TD children and children with CP. In Study 2, the amount of variability in retention is significantly negatively correlated with MaxZ (*R*^2^ = 0.373, *p* = 0.035) and with MeanZ (*R*^2^ = 0.293, *p* = 0.0216) at retention. The amount of variability in the transfer session is significantly negatively correlated with MaxZ (*R*^2^ = 0.336, *p* = 0.010), and MeanZ (*R*^2^ = 0.503, *p* < 0.001) at transfer, with no difference in this relationship between TD children and children with CP.

## Discussion

This secondary data analysis explored within- and between-child performance variability during practice of a novel postural control task in a non-immersive VE at acquisition, retention and transfer sessions in children with hemiplegic CP and TD children. Consistent with evidence for motor skill acquisition with practice in children with CP [e.g., (69–71)], performance on the task improved over repeated trials during the acquisition session, although it did not reach the same success level as TD children. We observed expected age differences in performance with older children outperforming younger children in each group. Performance decreased at the end of each practice session of our standing postural control tasks, especially for younger children with CP: e.g., from trial 19 to trial 20, the final trial of the acquisition stage, performance decreases by ≥0.2, enough for the error bars to exclude the mean curve in both CP age groups, but neither TD age group (Figure 3). Fatigue is one possible explanation for this observation. Children with CP demonstrate greater energy expenditure in ambulatory tasks as compared to TD children (72). Bronton and Bartlett (73) surveyed fatigue in 130 young adults with CP at all GMFCS levels, finding that while fatigue was highest in individuals at higher GMFCS levels (II-V), the majority (92%) of participants reported being fatigued at least a quarter of the day or more.

The amount of between-child variability in performance differed between groups, age groups and sessions. As hypothesized, younger children with CP had the highest between-child variability at each session, likely reflecting the known heterogeneity in motor abilities in children with CP compounded by a lesser amount of motor skill experience at this age. Contrary to our hypothesis, children with CP did not always demonstrate more between-child variability as compared to TD children. Patterns of variability in each session differed for older TD children and children with CP. Older children with CP had the least between-child variability in acquisition and transfer sessions but had highest between-child variability during retention. In contrast, TD children had the most variability in acquisition and the least variability in retention. This finding is explained by the fact that children with CP had consistently lower scores across participants.

The greater amount of between-child variability in retention in children with CP, combined with lower scores in the retention session, may reflect motor learning impairments in children with CP as compared to TD children, who more consistently retained task performance improvements. Information about children's postural control abilities obtained from baseline testing postural control abilities (Table 1) shows expected differences between TD children and children with CP due to motor impairment. However, postural control results were not especially heterogeneous among older children with CP, which further explains the low between-child variability. We did not collect detailed demographic data from children as to their current physical activity or sports participation that could help to elucidate between-child variability in task performance at retention in terms of movement experience. High between-child variability in all groups and age groups in the transfer session also suggests the importance of exploring other child factors that influence motor learning. For example, factors such as attention or motivation that were unmeasured here may illuminate between-child performance differences.

With respect to within-child variability, individual children were least variable in their performance across trials in the retention, suggesting that children achieved sufficient task competence to maintain stable performance after a period of no practice. As hypothesized, younger children demonstrated greater within-child variability than older children at each session; however, we were surprised to see lower within-child variability in children with CP as compared to TD children in acquisition and transfer. This finding may be explained again by the overall consistently poorer performance (i.e., lower scores) of children with CP as compared to TD children, limiting the range of scores across which subjects can vary. This is particularly true in the transfer session, which had the lowest scores for children with CP (especially for younger children). A less challenging task and a larger sample size may have resulted in greater information about within-child variability in performance over time. Simple prospective power calculations indicate that, for a balanced design, a sample size of 25 subjects per age group and developmental group would be required to detect our observed difference in the transfer session at alpha = 0.05. Subsequent studies can evaluate within-child variability over longer durations of practice, while considering the challenge of fatigue and motor endurance in this population.

Movement execution variability is one contributor to performance variability across repeated trials. In our studies, performance score was directly based on movement execution: the precision of controlling weight-shifting of the CoP over a static base of support. Studies involving repeated task practice of seated reaching tasks in non-immersive VEs demonstrate that children with CP reduce their movement execution variability with practice (16, 57, 74). However, these studies did not include retention or transfer tasks. In a game play situation, children with CP playing active video games in a non-immersive VE demonstrate within- and between-child variability at a movement execution level during repetitive game play (59, 60). This game play situation has some similarity to our study tasks in having greater opportunities for exploration in movement strategies as compared to studies involving restricted single arm reaching tasks in a seated position. This could have influenced the amount of between-child variability as children tried different strategies to achieve the task goal.

The relationship of within-child variability to performance differed between the 2 studies, with a positive correlation (greater variability in acquisition associated with better scores) in Study 1 and a negative correlation in Study 2. Both studies had similar movement requirements for success and similar visual feedback about how avatar position determined score. However, the visual display differed between the 2 studies, with the full path visible in a 3rd person perspective in Study 2 and the path emerging with movement in a first-person perspective in Study 1. Children with CP found Study 2 more challenging, as scores were lower as compared to Study 1. The path width was narrower in Study 2 as compared to study 1, leading to more penalty for increased variation in CoP position. In Study 1, we can speculate that with a slightly wider path that constantly revealed itself in a first person perspective, children had more tolerance for variation and that those who took advantage of this may have found a more optimal strategy that resulted in higher scores. Differences in the relationship between amount of variability and performance according to task requirements and VE visual display perspective suggest the need for subsequent research to explore the influence of these and other factors on variability.

Our variability metric did not enable us to partition variability into adaptive or error components. Using more sophisticated statistical models to understand the structure of variability can provide more insight into randomness and exploration patterns (31, 32). Example methods that could be useful for exploring the structure of different solutions in simple redundant tasks include the Tolerance, Noise and Covariation Approach (TNC), the Uncontrolled Manifold Approach, and the Goal-Equivalent Manifold approach; readers are directed to Sternad (32) for an overview. Of these, only the TNC method was purposefully developed to evaluate learning processes in changes in variability over time; however, it has not yet been applied to complex 3D tasks, as the model assumes task outcome redundancy from two precisely quantified input variables.

Exploring variability in movement execution during new task learning in non-immersive VEs through kinematic analyses can provide additional insight into this important source of performance variability. To understand how variability differs between virtual and physical environments, subsequent research can use within-participant designs to compare variability in the same task in a VE and an equivalent physical environment. An unexplored area of future research is whether VEs are relevant training paradigms to encourage development of variability/behavioral flexibility (75). Given that task features and task challenge can be precisely manipulated in a VE, these features could elicit practice of variable responses to differing task conditions and adapting to different task constraints. To achieve this goal, more knowledge about the similarity of movements in VEs to the physical environment and how learning transfers to the physical environment in children with CP is important to understand the degree of similarity required to facilitate transfer. Other hypothesized intertwined factors that might influence variability include children's attention, fatigue, effort, and motivation. Indeed, a predominant rationale for the use of VEs is that they elicit and sustain children's motivation and attention to participate in repetitive training (76).

This study has several limitations. Conclusions about variability in performance at retention and transfer sessions are limited by inconsistent rest periods between acquisition and retention/transfer sessions between participants, ranging from 2 to 7 days. These periods were necessary to accommodate family schedules in data collection. Our sample size was small and unbalanced, with the CP group having an especially low number of participants. While the mixed model approach utilized in the lme4 package is designed to be robust to unbalanced data (77), we interpret our results cautiously and would encourage follow-up studies with larger sample sizes.

## Conclusion

Performance variability can be an important source of information about differences in children's responses to interventions and should be considered in the design of rehabilitation protocols. This study is the first to specifically investigate performance variability over time during learning of standing postural control tasks in a non-immersive VE in children with hemiplegic CP. Findings contribute to the evidence base about differences in motor skill learning in children with CP as compared to TD peers in these novel intervention environments. Between- and within-child performance variability in children with CP is consistent with expected challenges with task performance due to motor impairments and age. A greater understanding of variability in motor skill learning in VEs is important to advance the debate as to the benefits and disadvantages of variability in motor skill learning and to understand whether the affordances of non-immersive VEs may make these interventions appropriate for training behavioral flexibility. Given that the relationship of within-child variability in skill acquisition differs according to the specific demands of the task, other factors that influence performance variability should be explored in subsequent studies, including differences in movement execution in VEs and cognitive factors such as attention and motivation. The design of VE-based interventions for children with hemiplegic CP can consider all these factors and their implications in order to maximize therapeutic benefit.

## Data Availability Statement

The raw data supporting the conclusions of this article will be made available by the authors, without undue reservation.

## Ethics Statement

The studies involving human participants were reviewed and approved by Northeastern University Institutional Review Board. Written informed consent to participate in this study was provided by the participants' legal guardian/next of kin. Written informed consent was obtained from the minor(s)' legal guardian/next of kin for the publication of any potentially identifiable images or data included in this article.

## Author Contributions

DL conceived of the study. MA and MC undertook the analyses and prepared the figures. MC, DL, and MA wrote and edited the manuscript. All authors agree to be accountable for the content of the work.

## Conflict of Interest

The authors declare that the research was conducted in the absence of any commercial or financial relationships that could be construed as a potential conflict of interest.
